# Supplementary material for: Bacterial lipid droplets bind to DNA via an intermediary protein that enhances survival under stress
Source: Nat Commun. 2017 Jul 6;8:15979. doi: 10.1038/ncomms15979 (PMC5504291; doi:10.1038/ncomms15979)
Supplement: Supplementary Information [file ncomms15979-s1.pdf]

Title of file for HTML: Supplementary Information
Description: Supplementary Figures and Supplementary Tables

Title of file for HTML: Supplementary Data 1
Description: Bacterial strains and plasmids used in this study.

Title of file for HTML: Supplementary Data 2
Description: Oligonucleotides used in this study.

Title of file for HTML: Peer Review File
Description:

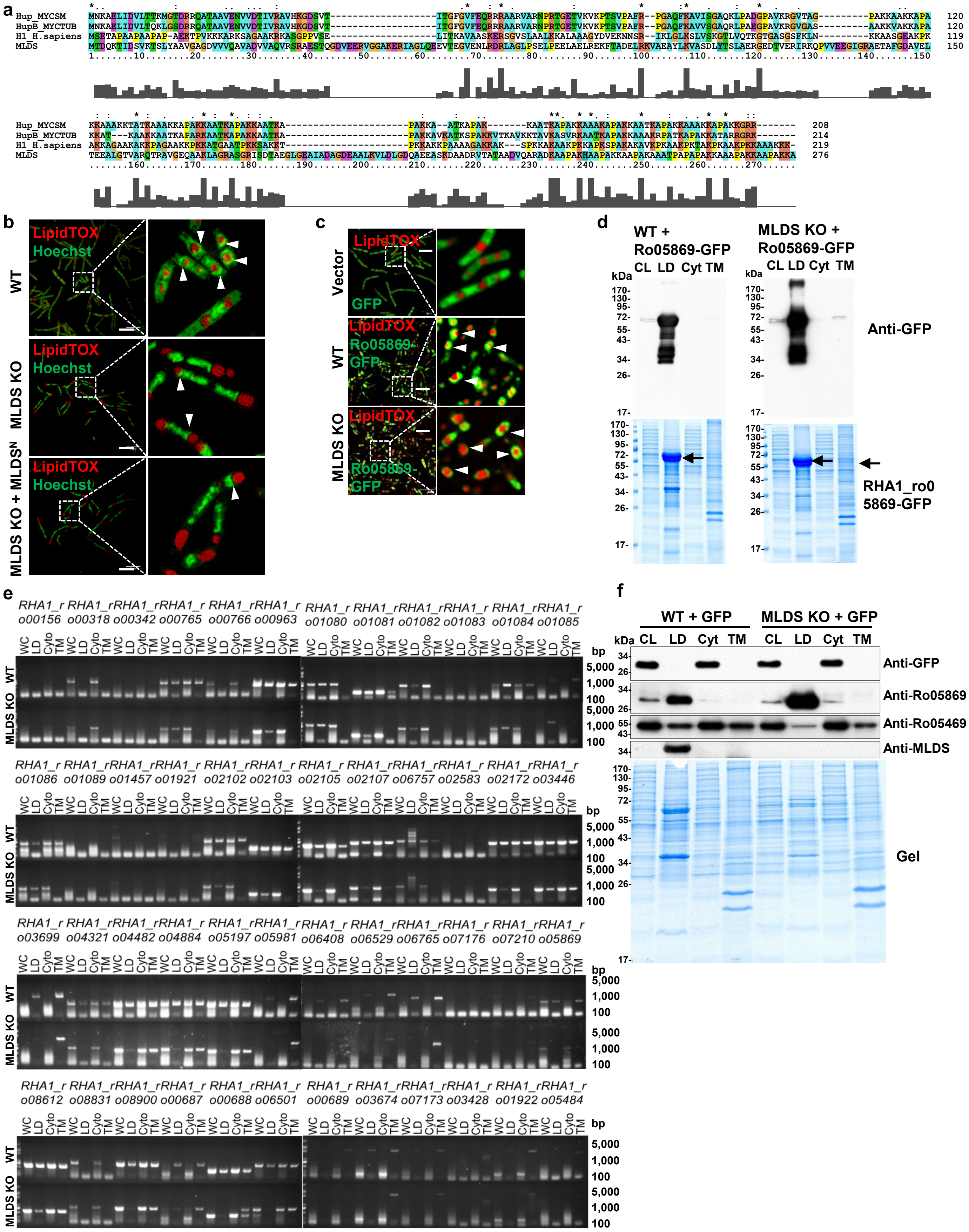
a
Hup_MYCSM
HupB_MYCTUB
H1_H.sapiens
MLDS
b
LipidTOX
Hoechst
WT
MLDS KO
MLDS KO + MLDS^N
c
Vector
WT
MLDS KO
GFP
Ro05869-GFP
d
WT + Ro05869-GFP
MLDS KO + Ro05869-GFP
CL LD Cyt TM
kDa
Anti-GFP
RHA1_ro05869-GFP
e
WC LD Cyto TM
WT
MLDS KO
bp
5,000
1,000
100
f
WT + GFP
MLDS KO + GFP
CL LD Cyt TM
Anti-GFP
Anti-Ro05869
Anti-Ro05469
Anti-MLDS
Gel

**Supplementary Figure 1: Lipid droplets bind genomic DNA by MLDS majorly in bacteria**

(**a**) MLDS contains sequence similarity with Hlp in mycobacteria and histone H1 in *Homo sapiens* by the ClustalX2 alignment.

(**b**) SIM images of wild type (WT), MLDS knockout (MLDS KO), and MLDS KO cells with overexpressed MLDS N-terminus ($MLDS^{N}$). The association of genomic DNA with LDs was indicated (white arrows). LDs and DNA were stained with LipidTOX red (red) and Hochest (green), respectively. Scale bar, 5 μm.

(**c**) Association of RHA1_ro05869 with LDs in WT and MLDS KO RHA1 was visualized by confocal microscopy. The LD targeting of RHA1-ro05869-GFP was indicated (white arrows) and the green signal appeared "ring-like" structure, which is in agreement with LD physiology. LDs were stained with LipidTOX red (red). Scale bar, 5 μm.

(**d**) Association of RHA1_ro05869 with LDs in WT and MLDS KORHA1 was determined by Western blotting analysis. RHA1_ro05869 was used as a LD marker in RHA1. CL, whole cell lysate; LD, lipid droplet; Cyto, cytosol; TM, total membrane.

(**e**) The PCR detection of selected genes in RHA1. 48 genes were detected by PCR using isolated LDs or other cell fractions as the template. WC, whole cell lysate; LD, lipid droplet; Cyt, cytosol; TM, total membrane.

(**f**) Western blot analysis of cell fractions of RHA1 WT + GFP and MLDS KO + GFP cells using anti-MLDS, anti-Ro05469, anti-Ro05869 and anti-GFP. CL, whole cell lysate; LD, lipid droplet; Cyt, cytosol; TM, total membrane. Gel was stained with Colloidal Blue. The experiment identified the purity of LD fraction, which is used for the control of Fig. 1f, 1g and supplementary fig. 1d, 1e.

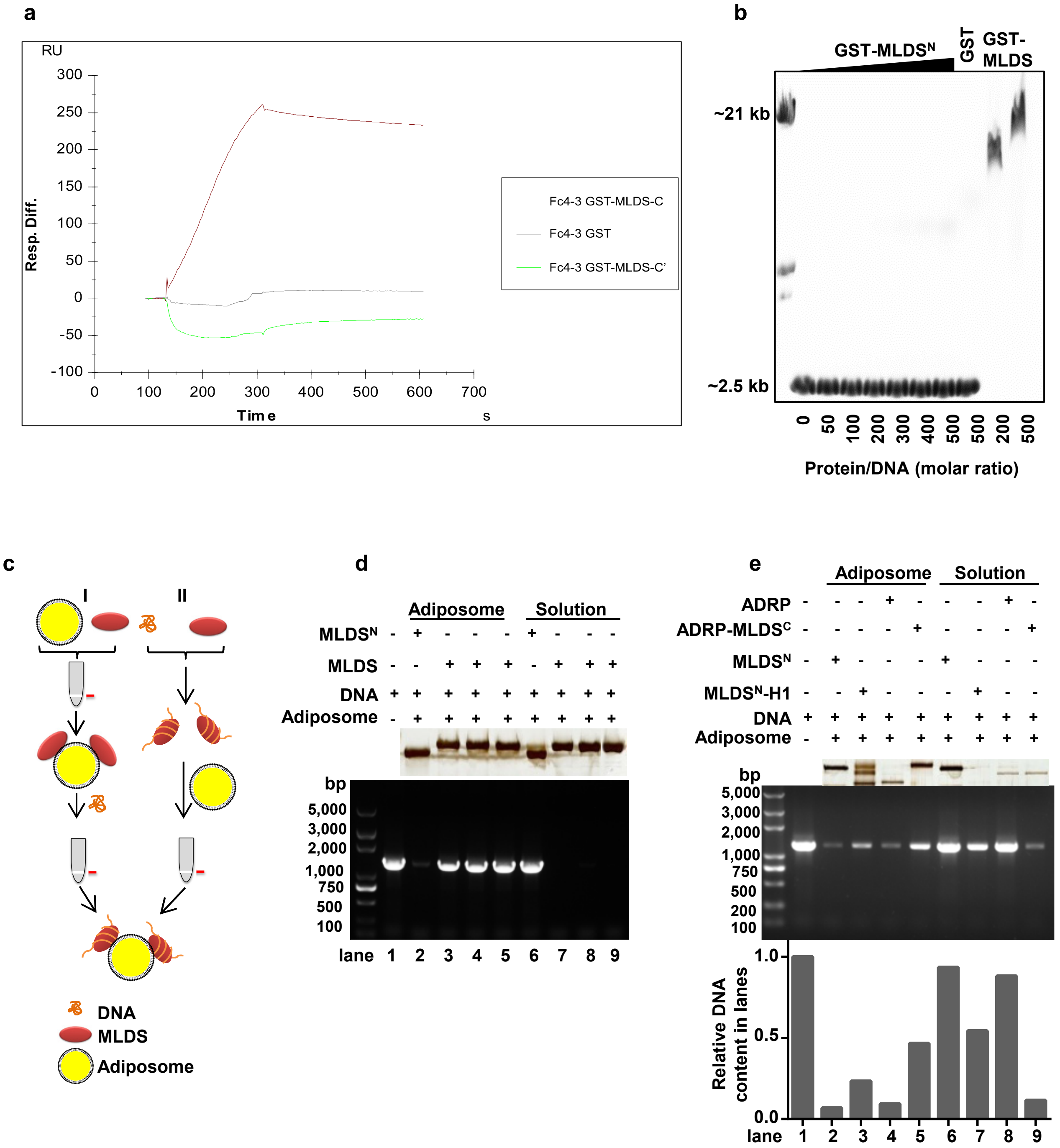

a
RU
300
250
200
150
100
50
0
-50
-100
Resp. Diff.
0
100
200
300
400
500
600
700
Time
s
Fc4-3 GST-MLDS-C
Fc4-3 GST
Fc4-3 GST-MLDS-C'
b
GST-MLDS$^N$
GST
GST-MLDS
~21 kb
~2.5 kb
0
50
100
200
300
400
500
500
200
500
Protein/DNA (molar ratio)
c
I
II
DNA
MLDS
Adiposome
d
Adiposome
Solution
MLDS$^N$
MLDS
DNA
Adiposome
bp
5,000
3,000
2,000
1,000
750
500
100
lane 1 2 3 4 5 6 7 8 9
e
Adiposome
Solution
ADRP
ADRP-MLDS$^C$
MLDS$^N$
MLDS$^N$-H1
DNA
Adiposome
bp
5,000
3,000
2,000
1,000
750
500
200
100
Relative DNA content in lanes
1.0
0.5
0.0
lane 1 2 3 4 5 6 7 8 9

**Supplementary Figure 2: MLDS C-terminus binds DNA *in vitro* by PAKKA motifs**

(**a**) SPR analysis of the interaction between $MLDS^{C}$ or $MLDS^{C'}$ and DNA. Biotin-DNA (about 1.5 kb) was coupled to the surface of a SA sensorchip. GST, GST-$MLDS^{C}$ or GST-$MLDS^{C'}$ was injected onto the DNA-coupled sensorchip. RU, resonance units.

(**b**) EMSA analysis of $MLDS^{N}$ and DNA. The 2.5 kb DNA was used for the assay and was stained by EB. The result revealed that $MLDS^{N}$ could not bind to DNA.

(**c, d**) Sketch (**c**) and validation (**d**) of adiposome binding assay using adiposomes, DNA, and MLDS or $MLDS^{N}$ by two mechanisms (I and II). I) Adiposomes and MLDS were incubated and isolated. The adiposome-bound MLDS was then incubated with DNA followed by re-isolation of adiposomes and analysis; II) DNA and MLDS were incubated and then the complex was incubated with adiposomes followed by re-isolation of the adiposomes and analysis. The results revealed that MLDS could recruit DNA to adiposomes in either condition. Mechanism I, lanes 4 and 8; Mechanism II, lanes 5 and 9.

(**e**) Adiposomes bind DNA by ADRP-$MLDS^{C}$ and $MLDS^{N}$-H1 fusion proteins. Validation of adiposome binding assays using adiposomes, DNA, and ADRP-$MLDS^{C}$ or $MLDS^{N}$-H1. DNA was detected by PCR and EB stained agarose gel (middle). The relative DNA content in the lanes was quantified using Image J software (bottom). Lane 1: DNA; lanes 2&6: $MLDS^{N}$, DNA and adiposomes; lanes 3&7: $MLDS^{N}$-H1, DNA and adiposomes; lanes 4&8: ADRP, DNA and adiposomes; lanes 5&9: ADRP-$MLDS^{C}$, DNA and adiposomes. Lanes 2-5 represented adiposome samples; lanes 6-9 represented solution samples. Protein was detected by sliver stain (top). ADRP-$MLDS^{C}$, $MLDS^{N}$ and $MLDS^{N}$-H1 represented GST-ADRP-$MLDS^{C}$, GST-$MLDS^{N}$ and GST-$MLDS^{N}$-H1, respectively.

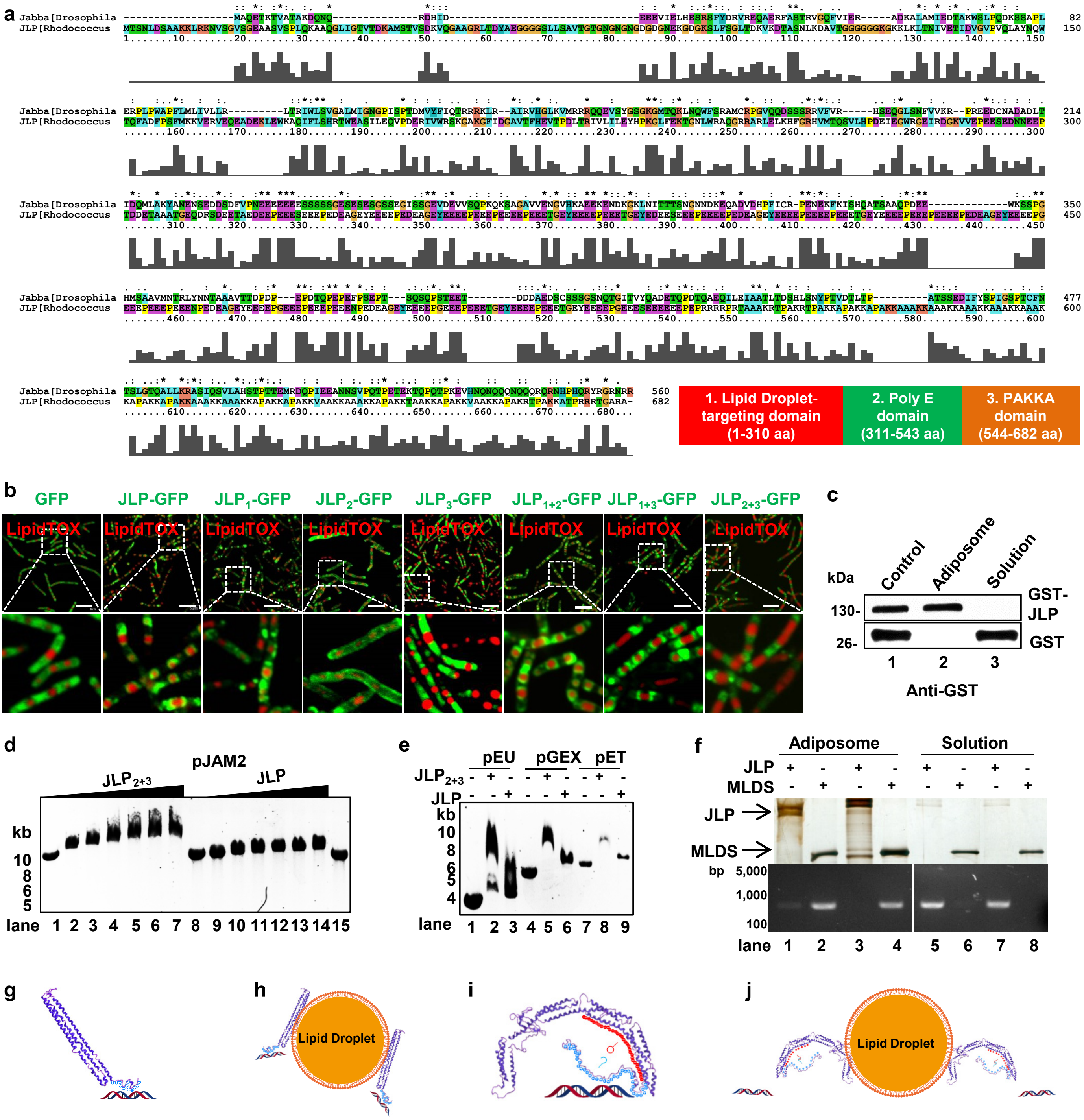

a
Jabba [Drosophila
JLP [Rhodococcus
1. Lipid Droplet-targeting domain (1-310 aa)
2. Poly E domain (311-543 aa)
3. PAKKA domain (544-682 aa)
b
GFP
JLP-GFP
$JLP_1$-GFP
$JLP_2$-GFP
$JLP_3$-GFP
$JLP_{1+2}$-GFP
$JLP_{1+3}$-GFP
$JLP_{2+3}$-GFP
LipidTOX
c
kDa
Control
Adiposome
Solution
130-
26-
GST-JLP
GST
1
2
3
Anti-GST
d
pJAM2
$JLP_{2+3}$
JLP
kb
10
8
6
5
lane 1 2 3 4 5 6 7 8 9 10 11 12 13 14 15
e
pEU
pGEX
pET
$JLP_{2+3}$
JLP
kb
10
8
6
5
4
lane 1 2 3 4 5 6 7 8 9
f
Adiposome
Solution
JLP
MLDS
bp
5,000
1,000
100
lane 1 2 3 4 5 6 7 8
g
h
Lipid Droplet
i
j
Lipid Droplet

**Supplementary Figure 3: Another lipid droplet-associated and PAKKA motif-contained protein, JLP cannot recruit DNA to lipid droplets**

(**a**) RHA1_ro00689 (JLP) contains sequence similarity with Jabba protein in *Drosophila* by the ClustalX2 alignment. The protein could be divided into 3 domains according to its sequence characteristic. The second and third domains are enriched in glutamic acid and lysine, respectively. The first domain was predicted to be LD-targeting domain.

(**b**) Confocal microscopy images of different JLP truncated mutants. The 1$^{st}$ domain of JLP was LD-targeting domain. WT cells with overexpression of different truncated mutants were cultivated in MSM to $OD_{600}$ = 2.0-2.5. Slides were imaged with an Olympus FV1000. Scale bar, 5 μm.

(**c**) Adiposome binding assay using adiposomes and JLP. Protein was detected by Western blot. 1: Protein; 2: adiposome fraction; 3: solution fraction.

(**d, e**) EMSA analysis of the interaction between JLP or $JLP_{2+3}$ and DNA. JLP (or without 1$^{st}$ domain) bound DNA without DNA sequence specificity. (**d**) The DNA is plasmid pJAM2-eGFP (about 10 kb). (**e**) JLP could bind some other plasmids (pEU, pGEX-6P-2, and pET-28a). Lanes 1, 4 and 7: DNA; lanes 2, 5 and 8: $JLP_{2+3}$ and DNA; lanes 3, 6 and 9: JLP and DNA.

(**f**) Validation of adiposome binding assay using adiposomes, DNA, and MLDS or JLP by two mechanisms (I and II). Mechanism I, lanes 1, 2, 5, and 6; Mechanism II, lanes 3, 4, 7, and 8. DNA was detected by PCR and EB stained agarose gel (bottom). Lanes 1&5: JLP and adiposomes were incubated and adiposomes were isolated, then DNA was incubated with JLP-bound adiposomes (mechanism I); lanes 2&6: MLDS and adiposomes were incubated and adiposomes were isolated, then DNA was incubated with MLDS-bound adiposomes (mechanism I); lanes 3&7: JLP and DNA were incubated first, and then adiposomes were added (mechanism II). lanes 4&8: MLDS and DNA were incubated first, and then adiposomes were added (mechanism II). lanes 1-4 represented adiposome samples; lanes 5-8 represented solution samples. Protein was detected by sliver staining (top). JLP and MLDS indicated GST-JLP and GST-MLDS.

(**g-j**) The 3D structure models of MLDS and JLP were predicted by I-TASSER. Both MLDS in solution (**g**) and on LDs (**h**) bound DNA. (**i**) JLP in solution could bind DNA by PAKKA motif. (**j**) JLP on LDs could not bind DNA because the PAKKA containing domain of JLP may interact with its poly E containing domain.

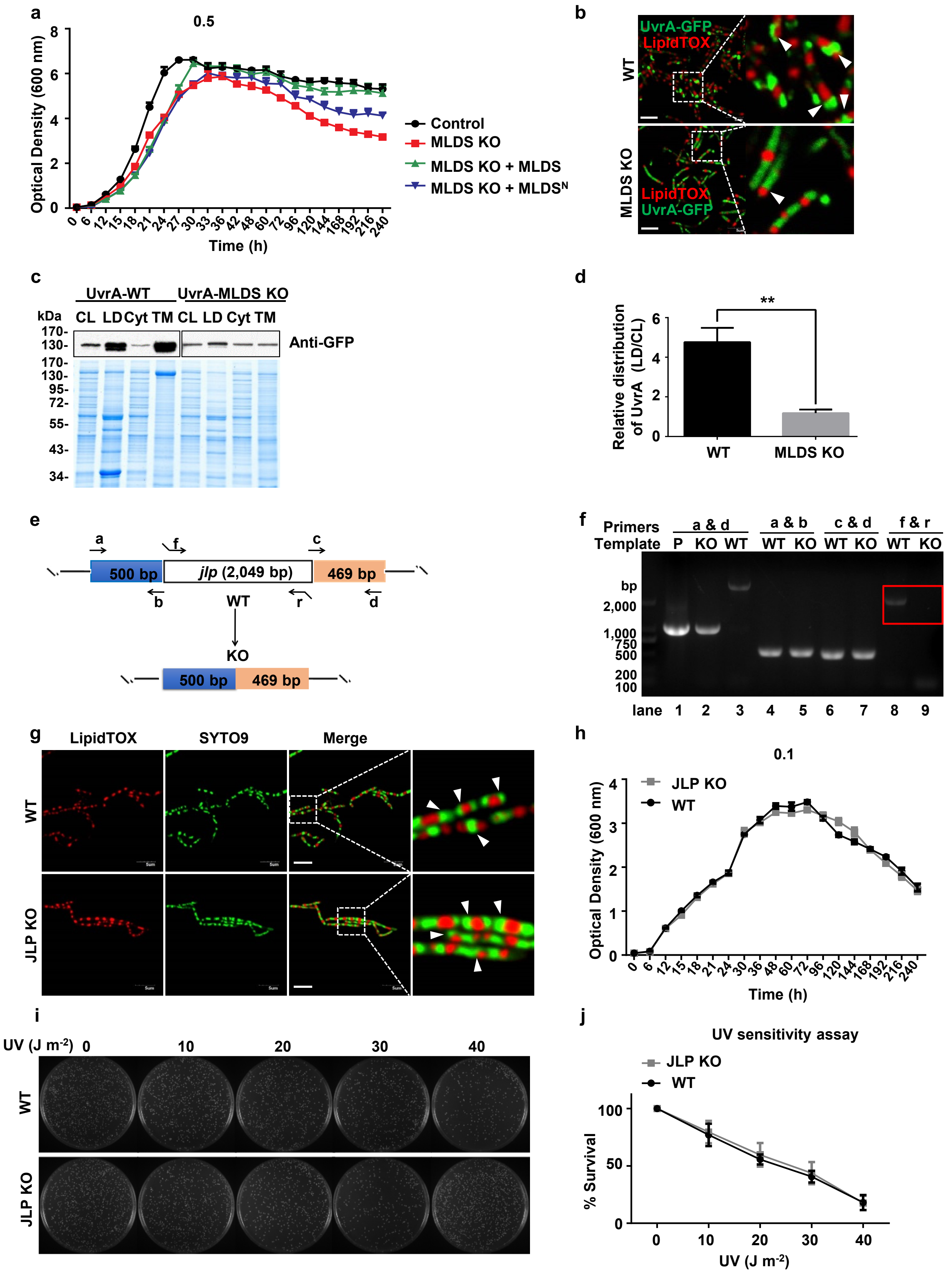

a
0.5
Optical Density (600 nm)
Time (h)
Control
MLDS KO
MLDS KO + MLDS
MLDS KO + MLDS^N
b
WT
MLDS KO
UvrA-GFP
LipidTOX
c
UvrA-WT
UvrA-MLDS KO
kDa
CL LD Cyt TM CL LD Cyt TM
170-
130-
95-
72-
55-
43-
34-
Anti-GFP
d
Relative distribution of UvrA (LD/CL)
**
WT
MLDS KO
e
a
f
c
500 bp
jlp (2,049 bp)
469 bp
b
WT
r
d
KO
f
Primers
Template
a & d
a & b
c & d
f & r
P KO WT WT KO WT KO WT KO
bp
2,000
1,000
750
500
200
100
lane 1 2 3 4 5 6 7 8 9
g
LipidTOX
SYTO9
Merge
WT
JLP KO
h
0.1
JLP KO
WT
Optical Density (600 nm)
Time (h)
i
UV (J m-2) 0 10 20 30 40
WT
JLP KO
j
UV sensitivity assay
JLP KO
WT
% Survival
UV (J m-2)

**Supplementary Figure 4: Deletion of JLP has no effect on binding of DNA to lipid droplets and bacterial survival in extreme conditions.**

(**a**) The growth curve of control WT, MLDS KO, MLDS KO + MLDS and MLDS KO + $MLDS^N$ strains in MSM with 0.5 g $L^{-1}$ NH4Cl. Data represent mean ± SD, n = 3.

(**b-d**) The distribution of UvrA-GFP in WT and MLDS KO cells. (**b**) Confocal microscopy images of UvrA-GFP location in WT and MLDS KO cells. The association of UvrA-GFP with LDs was indicated (white arrows). LDs were stained with LipidTOX red (red). Scale bar, 5 μm. (**c**) The cell fraction analysis of UvrA-GFP localization by Western blot. (**d**) Quantification of the ratio of UvrA-GFP in LD fraction to CL fraction in WT and MLDS KO cells in (**c**). Data represent mean ± SEM, n = 3. *P < 0.05, two-tailed *t*-test.

(**e**) Sketch of homologous recombination knockout *jlp* gene.

(**f**) Identification of *jlp* gene deletion mutant by PCR. M, marker; lane 1, positive control of *jlp* gene PCR fragment using primers a/d with knockout pK18mobsacB plasmid as template; lane 2, the PCR fragment of *jlp* gene in deletion mutant was about 969 bp, the same as positive control; lane 3, the WT gave a size of about 3,000 bp; lane 4-5, the left flank sequences of AB were 500 bp using primers a/b with WT and deletion mutant cells as templates, respectively; lane 6-7, the right flank sequences of CD were 469 bp using primers c/d with the same templates as lane 4-5; lane 8-9, to further confirmed the deletion of target gene, f and r primers in *jlp* gene sequence was used. The target gene fragment was only present in WT of lane 8 while not in *jlp* deletion mutant of lane 9. Each primer used was shown in (**e**).

(**g**) Confocal microscopy images of WT and JLP KO cells. JLP deletion had no effect on DNA association with LDs. The association of genomic DNA with LDs was indicated (white arrows). LDs and DNA were stained with LipidTOX red (red) and SYTO9 (green). Scale bar, 5 μm.

(**h**) The growth curve of WT and JLP KO strains in extremely lacking nitrogen source medium (MSM with 0.1g $L^{-1}$ $NH_4Cl$). No significant growth difference between JLP KO cells and WT cells was observed. Data represent mean ± SD, n = 3.

(**i, j**) UV sensitivity assay. No significant difference between JLP KO cells and WT cells after UV exposure was detected. WT and JLP KO strains were cultivated after UV treatment (0, 10, 20, 30 and 40 J $m^{-2}$), in triplicates. Data represent mean ± SEM, n = 3 (**i**). The bacteria growth situation was shown in (**j**).

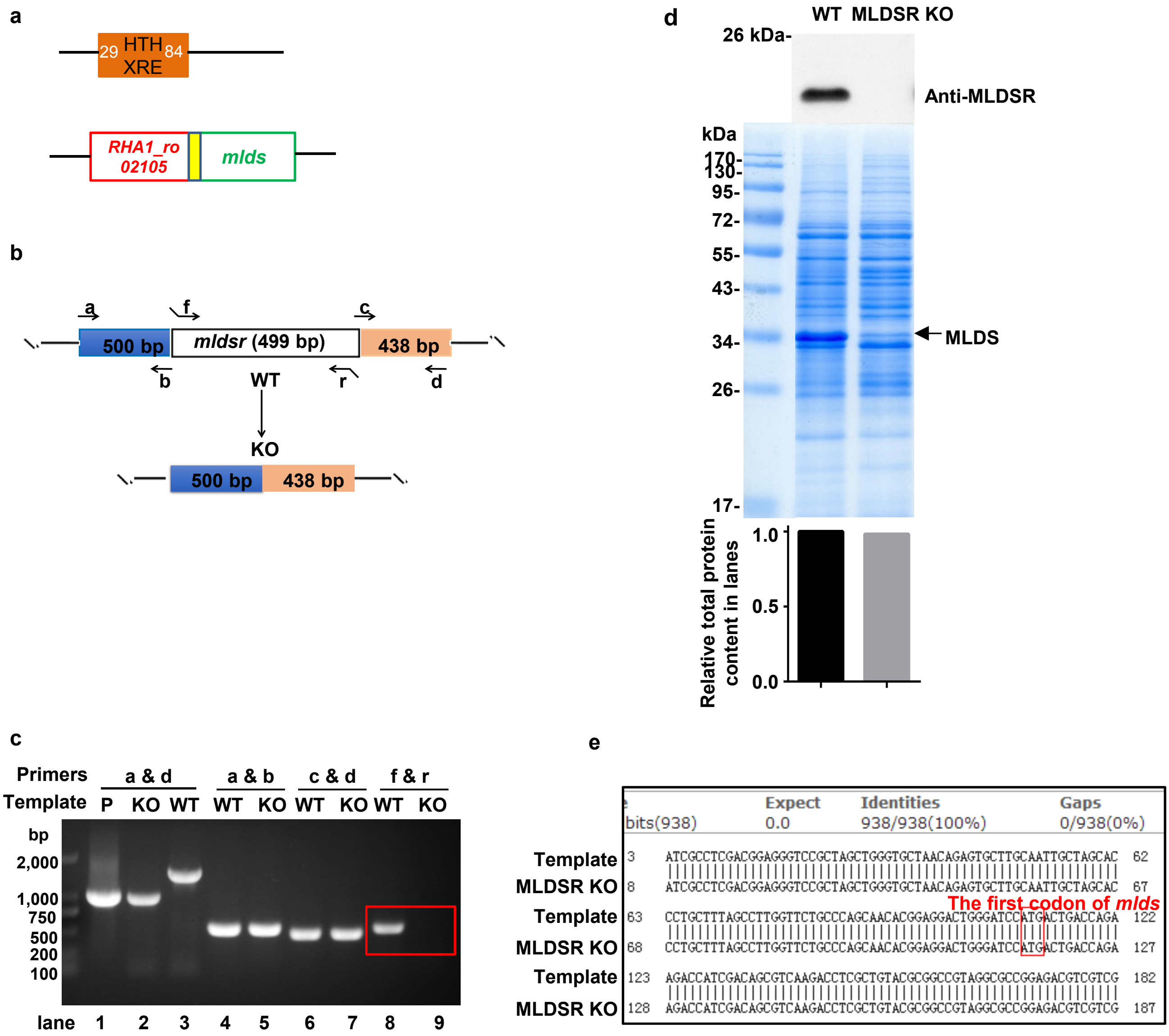
a
29
HTH
XRE
84
RHA1_ro
02105
mlds
b
a
f
c
500 bp
mldsr (499 bp)
438 bp
b
WT
r
d
KO
500 bp
438 bp
c
Primers
a & d
a & b
c & d
f & r
Template
P
KO
WT
WT
KO
WT
KO
WT
KO
bp
2,000
1,000
750
500
200
100
lane
1
2
3
4
5
6
7
8
9
d
WT
MLDSR KO
26 kDa-
Anti-MLDSR
kDa
170-
130-
95-
72-
55-
43-
34-
26-
17-
MLDS
Relative total protein content in lanes
1.0
0.5
0.0
e
Expect
Identities
Gaps
bits(938)
0.0
938/938(100%)
0/938(0%)
Template 3 ATCGCCTCGACGGAGGGTCCGCTAGCTGGGTGCTAACAGAGTGCTTGCAATTGCTAGCAC 62
MLDSR KO 8 ATCGCCTCGACGGAGGGTCCGCTAGCTGGGTGCTAACAGAGTGCTTGCAATTGCTAGCAC 67
The first codon of mlds
Template 63 CCTGCTTTAGCCTTGGTTCTGCCCAGCAACACGGAGGACTGGGATCCATGACTGACCAGA 122
MLDSR KO 68 CCTGCTTTAGCCTTGGTTCTGCCCAGCAACACGGAGGACTGGGATCCATGACTGACCAGA 127
Template 123 AGACCATCGACAGCGTCAAGACCTCGCTGTACGCGGCCGTAGGCGCCGGAGACGTCGTCG 182
MLDSR KO 128 AGACCATCGACAGCGTCAAGACCTCGCTGTACGCGGCCGTAGGCGCCGGAGACGTCGTCG 187

**Supplementary Figure 5: Analysis of *mldsr* deletion mutant**

(**a**) Diagrams of RHA1_ro02105 N-terminal predicted xenobiotic response element (XRE) helix-turn-helix (HTH) DNA-binding motif (top). Genes *RHA1_ro02105* and *mlds* are in the same operon in the RHA1 genome (bottom).

(**b**) Sketch of homologous recombination knockout *RHA1_ro02105* gene.

(**c**) Identification of *RHA1_ro02105* gene deletion mutant by PCR. M, marker; lane 1, positive control of *RHA1_ro02105* gene PCR fragment using primers a/d with knockout pK18mobsacB plasmid as template; lane 2, the PCR fragment of *RHA1_ro02105* gene in deletion mutant was about 938 bp, the same as positive control; lane 3, the WT gave a size of about 1,437 bp; lane 4-5, the left flank sequences of AB were 500 bp using primers a/b with WT and deletion mutant cells as templates, respectively; lane 6-7, the right flank sequences of CD were 438 bp using primers c/d with the same templates as lane 4-5; lane 8-9, to further confirmed the deletion of target gene, f and r primers in *RHA1_ro02105* gene sequence was used. The target gene fragment was only present in WT of lane 8 while not in *RHA1_ro02105* deletion mutant of lane 9. Each primer used was shown in (**b**).

(**d**) Identification of RHA1_ro02105 protein knockout using Western blot. Top panel: Western blot using anti-RHA1_ro02105, middle panel: gel stained with Colloidal Blue, bottom panel: the relative total protein content in the lanes through quantification using Image J software. MLDS expression decreased.

(**e**) DNA sequence alignment of the deletion mutant strain PCR products (primers a/d) (MLDSR KO) and published genome in RHA1 (Template) using BLAST in NCBI database. The first codon of mlds gene is shown (red frame). The deletion of *RHA1_ro02105* was specific. There was no frame shift in *mlds* gene, which suggests that the *cis*-element and gene region of *mlds* in MLDSR KO cells are intact.

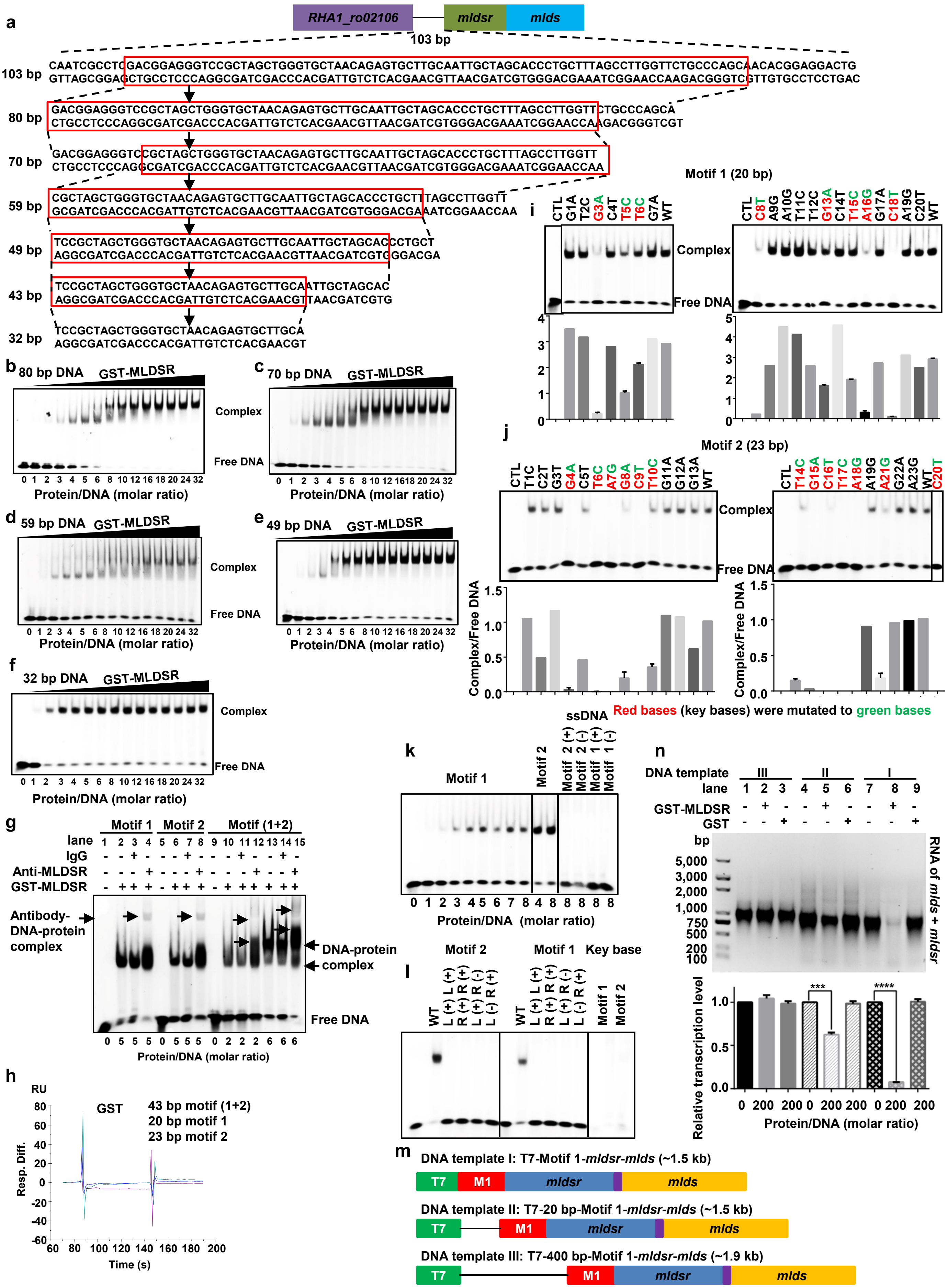

a
RHA1_ro02106
mldsr
mlds
103 bp
103 bp
CAATCGCCTCGACGGAGGGTCCGCTAGCTGGGTGCTAACAGAGTGCTTGCAATTGCTAGCACCCTGCTTTAGCCTTGGTTCTGCCCAGCAACACGGAGGACTG
GTTAGCGGAGCTGCCTCCCAGGCGATCGACCCACGATTGTCTCACGAACGTTAACGATCGTGGGACGAAATCGGAACCAAGACGGGTCGTTGTGCCTCCTGAC
80 bp
GACGGAGGGTCCGCTAGCTGGGTGCTAACAGAGTGCTTGCAATTGCTAGCACCCTGCTTTAGCCTTGGTTCTGCCCAGCA
CTGCCTCCCAGGCGATCGACCCACGATTGTCTCACGAACGTTAACGATCGTGGGACGAAATCGGAACCAAGACGGGTCGT
70 bp
GACGGAGGGTCCGCTAGCTGGGTGCTAACAGAGTGCTTGCAATTGCTAGCACCCTGCTTTAGCCTTGGTT
CTGCCTCCCAGGCGATCGACCCACGATTGTCTCACGAACGTTAACGATCGTGGGACGAAATCGGAACCAA
59 bp
CGCTAGCTGGGTGCTAACAGAGTGCTTGCAATTGCTAGCACCCTGCTTTAGCCTTGGTT
GCGATCGACCCACGATTGTCTCACGAACGTTAACGATCGTGGGACGAAATCGGAACCAA
49 bp
TCCGCTAGCTGGGTGCTAACAGAGTGCTTGCAATTGCTAGCACCCTGCT
AGGCGATCGACCCACGATTGTCTCACGAACGTTAACGATCGTGGGACGA
43 bp
TCCGCTAGCTGGGTGCTAACAGAGTGCTTGCAATTGCTAGCAC
AGGCGATCGACCCACGATTGTCTCACGAACGTTAACGATCGTG
32 bp
TCCGCTAGCTGGGTGCTAACAGAGTGCTTGCA
AGGCGATCGACCCACGATTGTCTCACGAACGT
b 80 bp DNA GST-MLDSR
c 70 bp DNA GST-MLDSR
d 59 bp DNA GST-MLDSR
e 49 bp DNA GST-MLDSR
f 32 bp DNA GST-MLDSR
Complex
Free DNA
0 1 2 3 4 5 6 8 10 12 16 18 20 24 32
Protein/DNA (molar ratio)
g
lane 1 2 3 4 5 6 7 8 9 10 11 12 13 14 15
Motif 1
Motif 2
Motif (1+2)
IgG
Anti-MLDSR
GST-MLDSR
Antibody-DNA-protein complex
DNA-protein complex
Free DNA
0 5 5 5 0 5 5 5 0 2 2 2 6 6 6
Protein/DNA (molar ratio)
h
RU
Resp. Diff.
GST
43 bp motif (1+2)
20 bp motif 1
23 bp motif 2
Time (s)
i
Motif 1 (20 bp)
CTL G1A T2C G3A C4T T5C T6C G7A WT
CTL C8T A9G A10G T11C T12C G13A C14T T15C A16G G17A C18T A19G C20T WT
Complex
Free DNA
j
Motif 2 (23 bp)
CTL T1C C2T G3T G4A C5T T6C A7G G8A C9T T10C G11A G12A G13A WT
CTL T14C G15A C16T T17C A18G A19G A21G G22A A23G WT C20T
Complex
Free DNA
Complex/Free DNA
Red bases (key bases) were mutated to green bases
k
Motif 1
Motif 2
ssDNA
Motif 2 (+)
Motif 2 (-)
Motif 1 (+)
Motif 1 (-)
0 1 2 3 4 5 6 7 8 4 8 8 8 8 8
Protein/DNA (molar ratio)
l
Motif 2
Motif 1
Key base
WT
L (+) L (+)
R (+) R (+)
L (+) R (-)
L (-) R (+)
Motif 1
Motif 2
m
DNA template I: T7-Motif 1-mldsr-mlds (~1.5 kb)
DNA template II: T7-20 bp-Motif 1-mldsr-mlds (~1.5 kb)
DNA template III: T7-400 bp-Motif 1-mldsr-mlds (~1.9 kb)
T7
M1
mldsr
mlds
n
DNA template III II I
lane 1 2 3 4 5 6 7 8 9
GST-MLDSR
GST
bp
5,000
3,000
2,000
1,000
750
500
200
100
RNA of mlds + mldsr
Relative transcription level
***
****
0 200 200 0 200 200 0 200 200
Protein/DNA (molar ratio)

**Supplementary Figure 6: MLDSR bind two adjacent DNA motifs**

(**a**) Sketch of sequentially shortening DNA sequence in length from 103 bp, 80 bp, 70 bp, 59 bp, 49 bp, 43 bp to 32 bp for identifying MLDSR binding box.

(**b-f**) EMSA analyses of MLDSR binding to the DNA motifs (80 bp, 70 bp, 59 bp, 49 bp and 32 bp). There were two bands representing DNA and protein complex from 103 bp to 43 bp, but not 32 bp.

(**g**) Super-EMSA analysis of the interaction between MLDSR and its binding motifs. Lanes 1, 5 and 9: only DNA; lanes 2, 6, 10 and 13: MLDSR and DNA; lanes 3, 7, 11 and 14: IgG, MLDSR and DNA; lanes 4, 8, 12 and 15: anti-MLDSR, MLDSR and DNA. DNA: motif 1 (lanes 1-4), motif 2 (lanes 5-8) and motif (1 + 2) (lanes 9-15). *(red) represents antibody-protein-DNA complex.

(**h**) SPR analysis using GST as the negative control. GST could not interact with motif (1 + 2), motif 2 and motif 1. Biotin-DNA (motif (1 + 2), motif 2 and motif 1) were coupled to the surface of a SA sensorchip. GST was injected onto the DNA-coupled sensorchip. RU, resonance units.

(**i, j**) Constitutive mutation analysis of motif 1 (**i**) and motif 2 (**j**). The EMSAs (top) and quantification (bottom) revealed the key base pairs (red) for MLDSR binding. Data represent mean ± SEM, n = 3.

(**k, l**) Characteristics of the interaction between MLDSR and motif 1 or motif 2 by using EMSA analysis. (**k**) The specificity of the interaction between MLDSR and motif 1 was higher than motif 2. MLDSR bound double-strand DNA, but not single-strand DNA. (**l**) The interaction between MLDSR and motif 1 or motif 2 required the directivity and space distance of DNA. EMSAs between MLDSR and DNA with different combinations including left (+) left (+) DNA, right (+) right (+) DNA, left (+) right (-) DNA, left (-) right (+) DNA and wild type - left (+) right (+) DNA revealed that MLDSR only bound to the wild type motif, but not to any directivity changed motifs. EMSAs between MLDSR and DNA with only key bases showed that MLDSR could not bind DNA with only key bases without other bases.

(**m**) Sketch of three DNA templates of *in vitro* transcription assays: I) Promoter and motif 1 were adjacent, II) There were ~20 bp between the promoter and motif 1, III) There were ~400 bp between the promoter and motif 1

(**n**) The *in vitro* transcription assay using 3 DNA templates. The transcriptional regulation of MLDSR occurs at the transcriptional initiation. RNA was detected by EB stained agarose gel (top). Quantification was performed using Image J software (bottom). Data represent mean ± SEM, n = 3. ***$P < 0.001$, ****$P < 0.0001$, two-tailed *t*-test.

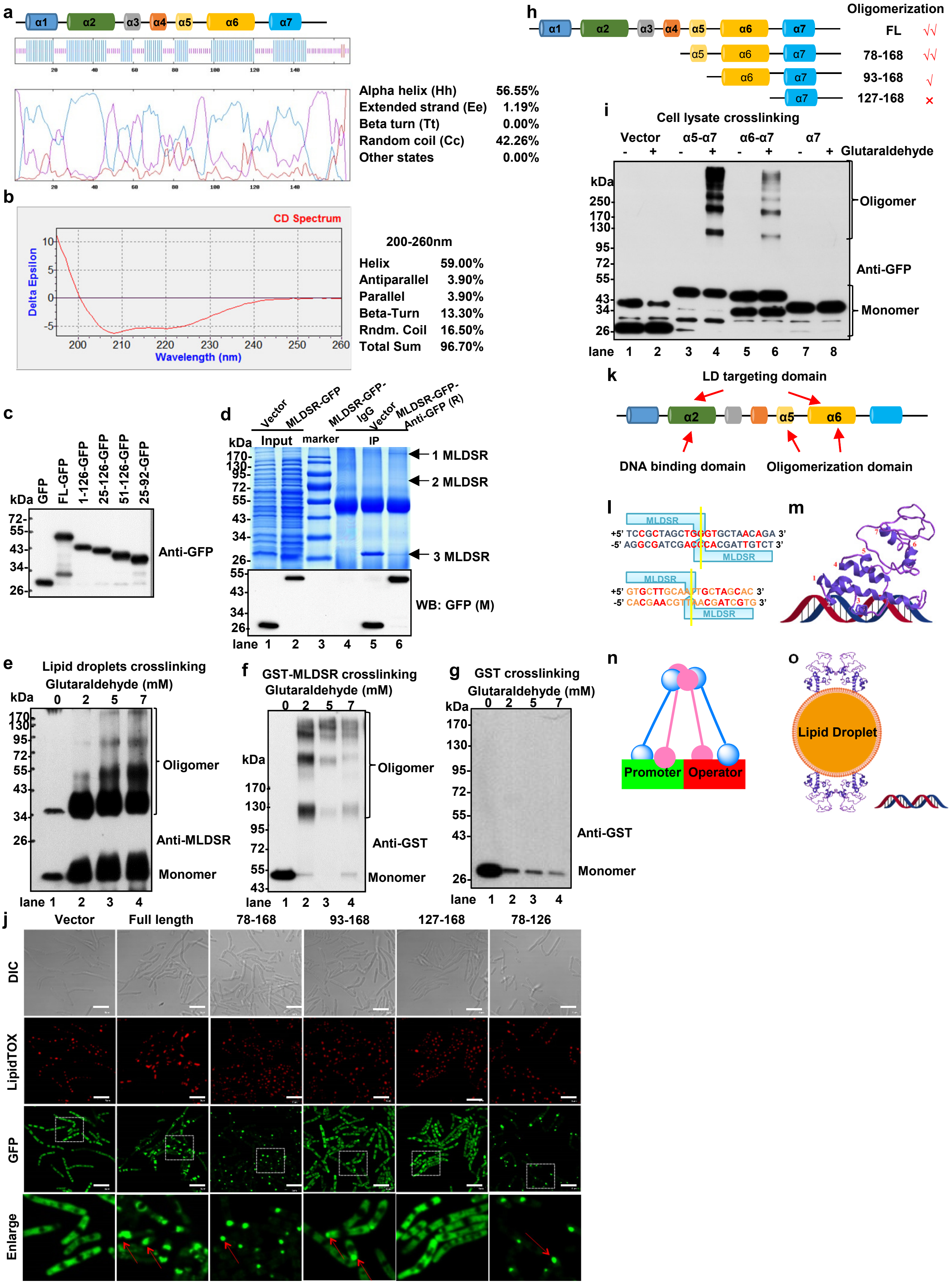
a
Alpha helix (Hh) 56.55%
Extended strand (Ee) 1.19%
Beta turn (Tt) 0.00%
Random coil (Cc) 42.26%
Other states 0.00%
b
CD Spectrum
Delta Epsilon
Wavelength (nm)
200-260nm
Helix 59.00%
Antiparallel 3.90%
Parallel 3.90%
Beta-Turn 13.30%
Rndm. Coil 16.50%
Total Sum 96.70%
c
GFP
FL-GFP
1-126-GFP
25-126-GFP
51-126-GFP
25-92-GFP
Anti-GFP
d
Vector
MLDSR-GFP
MLDSR-GFP-IgG
Vector
MLDSR-GFP-Anti-GFP (R)
Input
marker
IP
1 MLDSR
2 MLDSR
3 MLDSR
WB: GFP (M)
e
Lipid droplets crosslinking
Glutaraldehyde (mM)
Oligomer
Anti-MLDSR
Monomer
f
GST-MLDSR crosslinking
Glutaraldehyde (mM)
Oligomer
Anti-GST
Monomer
g
GST crosslinking
Glutaraldehyde (mM)
Anti-GST
Monomer
h
Oligomerization
FL
78-168
93-168
127-168
i
Cell lysate crosslinking
Vector
α5-α7
α6-α7
α7
Glutaraldehyde
Oligomer
Anti-GFP
Monomer
j
Vector
Full length
78-168
93-168
127-168
78-126
DIC
LipidTOX
GFP
Enlarge
k
LD targeting domain
DNA binding domain
Oligomerization domain
l
MLDSR
+5' TCCGCTAGCTGGGTGCTAACAGA 3'
-5' AGGCGATCGACCCACGATTGTCT 3'
+5' GTGCTTGCAATTGCTAGCAC 3'
-5' CACGAACGTTAACGATCGTG 3'
m
n
Promoter
Operator
o
Lipid Droplet

**Supplementary Figure 7: The structure and function characteristic of MLDSR**

(**a, b**) Secondary structure of MLDSR was predicted using the Network Protein Sequence Analysis (NPSA) (https://npsa-prabi.ibcp.fr) with GOR4 method (**a**) and circular dichroism (CD) experiment (**b**). There were 7 α-helices in MLDSR.

(**c**) Western blot analysis of different truncation mutants fused with GFP in bacteria by anti-GFP.

(**d**) CoIP-MS analysis of MLDSR-GFP overexpressed RHA1 cells using anti-GFP. The bands indicated by arrows were cut for MS identification.

(**e**) Glutaraldehyde crosslinking and Western blot analysis of isolated LDs from RHA1 by anti-MLDSR. Lanes 1-4: concentration of glutaraldehyde was 0, 2, 5, and 7 mM.

(**f, g**) GST-MLDSR (**f**), but not GST (**g**), could form oligomer *in vitro* by glutaraldehyde crosslinking. Purified GST-MLDSR and GST proteins from *E.coli* in the presence of glutaraldehyde were examined by Western blot with anti-GST. Lanes 1-4: concentration of glutaraldehyde was 0, 2, 5, and 7 mM.

(**h**) Sketch of the truncated mutant proteins (78-168, 93-168, 127-168 and 78-126 amino acids).

(**i**) Glutaraldehyde crosslinking and Western blot analysis of different truncated mutants by anti-GFP. Concentration of glutaraldehyde was 2 mM.

(**j**) Confocal microscopy images of different MLDSR truncated mutants. The oligomer seemed to be green spot (red arrows) in the images. Scale bar, 5 μm.

(**k**) Architecture summary of the lipid droplet-targeting, DNA binding domain, and oligomerization domain of MLDSR. LD targeting domain of MLDSR is helices α2 and α6, DNA binding domain is helix α2, and oligomerization domain is helices α5 and α6.

(**l-o**) The molecular mechanism of MLDSR regulating transcription. MLDSR bound promoter and operator (**l**).The 3D structure of MLDSR was predicted by I-TASSER (**m**). MLDSR could bind operator (and promoter) by oligomerization (**n**). Oligomeric LD-associated MLDSR could not bind DNA (**o**). These findings that the LD localization region of MLDSR was helices α2 and α6, motif 1 and motif 2 of MLDSR binding were symmetrical, and MLDSR formed oligomers by helices α5 and α6 suggest that oligomerization of the protein was required for its LD location and DNA binding.

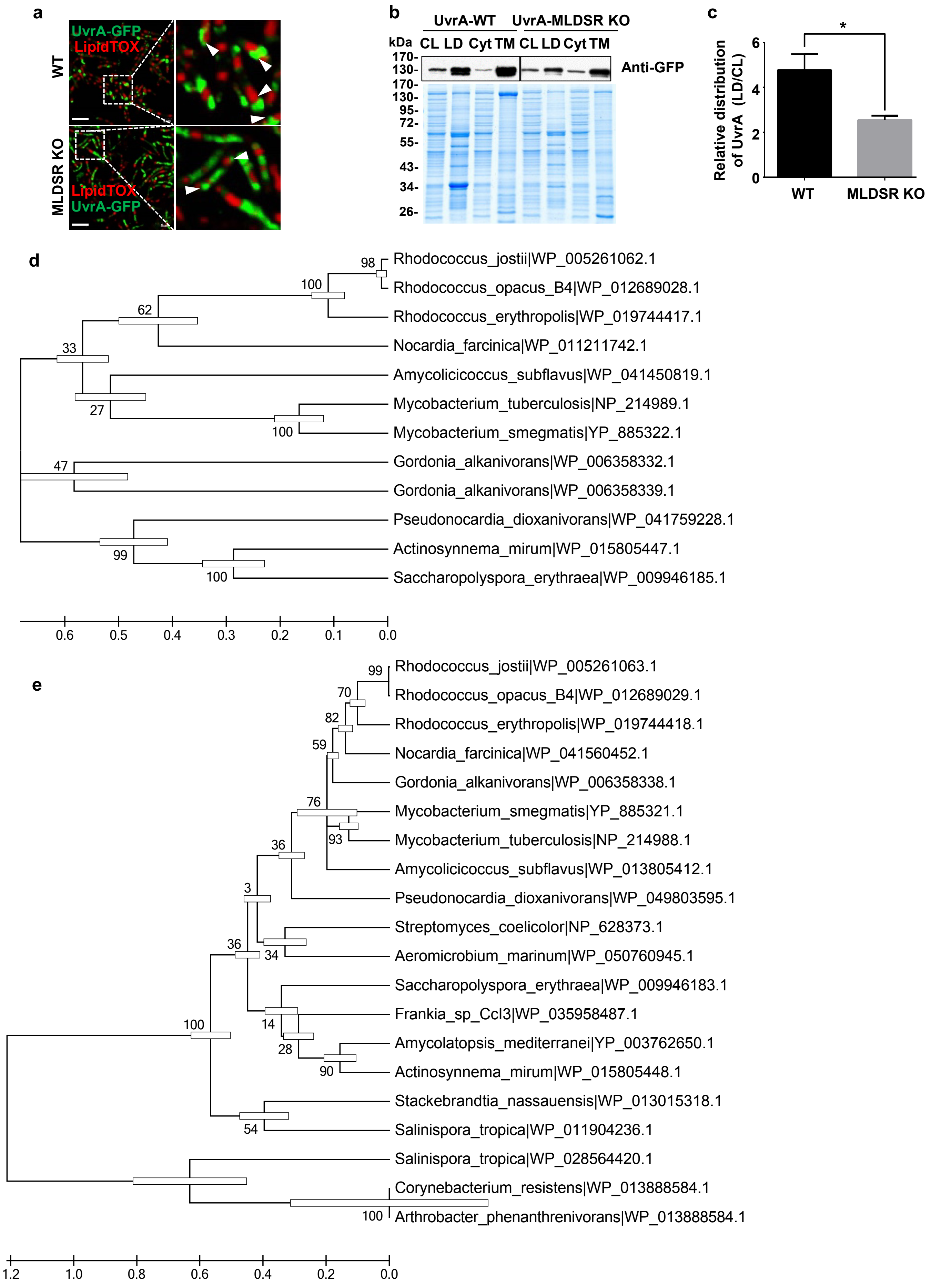
a
WT
MLDSR KO
UvrA-GFP
LipidTOX
b
UvrA-WT
UvrA-MLDSR KO
kDa
CL LD Cyt TM CL LD Cyt TM
170-
130-
Anti-GFP
170-
130-
95-
72-
55-
43-
34-
26-
c
*
Relative distribution of UvrA (LD/CL)
6
4
2
0
WT
MLDSR KO
d
Rhodococcus_jostii|WP_005261062.1
Rhodococcus_opacus_B4|WP_012689028.1
Rhodococcus_erythropolis|WP_019744417.1
Nocardia_farcinica|WP_011211742.1
Amycolicicoccus_subflavus|WP_041450819.1
Mycobacterium_tuberculosis|NP_214989.1
Mycobacterium_smegmatis|YP_885322.1
Gordonia_alkanivorans|WP_006358332.1
Gordonia_alkanivorans|WP_006358339.1
Pseudonocardia_dioxanivorans|WP_041759228.1
Actinosynnema_mirum|WP_015805447.1
Saccharopolyspora_erythraea|WP_009946185.1
98
100
62
33
27
100
47
99
100
0.6
0.5
0.4
0.3
0.2
0.1
0.0
e
Rhodococcus_jostii|WP_005261063.1
Rhodococcus_opacus_B4|WP_012689029.1
Rhodococcus_erythropolis|WP_019744418.1
Nocardia_farcinica|WP_041560452.1
Gordonia_alkanivorans|WP_006358338.1
Mycobacterium_smegmatis|YP_885321.1
Mycobacterium_tuberculosis|NP_214988.1
Amycolicicoccus_subflavus|WP_013805412.1
Pseudonocardia_dioxanivorans|WP_049803595.1
Streptomyces_coelicolor|NP_628373.1
Aeromicrobium_marinum|WP_050760945.1
Saccharopolyspora_erythraea|WP_009946183.1
Frankia_sp_CcI3|WP_035958487.1
Amycolatopsis_mediterranei|YP_003762650.1
Actinosynnema_mirum|WP_015805448.1
Stackebrandtia_nassauensis|WP_013015318.1
Salinispora_tropica|WP_011904236.1
Salinispora_tropica|WP_028564420.1
Corynebacterium_resistens|WP_013888584.1
Arthrobacter_phenanthrenivorans|WP_013888584.1
99
70
82
59
76
93
36
3
36
34
100
14
28
90
54
100
1.2
1.0
0.8
0.6
0.4
0.2
0.0

**Supplementary Figure 8: The phylogenetic tree of the orthologous proteins**

(**a**-**c**) The distribution of UvrA-GFP in WT and MLDSR KO cells. (**a**) Confocal microscopy images of UvrA-GFP location in WT and MLDSR KO cells. The association of UvrA-GFP with LDs was indicated (white arrows). LDs were stained with LipidTOX red (red). Scale bar, 5 μm. (**b**) The cell fraction analysis of UvrA-GFP localization by Western blot. (**c**) Quantification of the ratio of UvrA-GFP in LD fraction to CL fraction in WT and MLDSR KO cells in (**b**). Data represent mean ± SEM, n = 3. *P < 0.05, two-tailed *t*-test.

(**d**, **e**) Best maximum likelihood tree for the orthologous proteins of MLDS (**d**) and MLDSR (**e**) in 20 taxa. The field before the '|' character is the uniq taxon code and the field after the '|' character is the uniq NCBI refseq protein accession ID.

**Figure 3b** Anti-MLDS

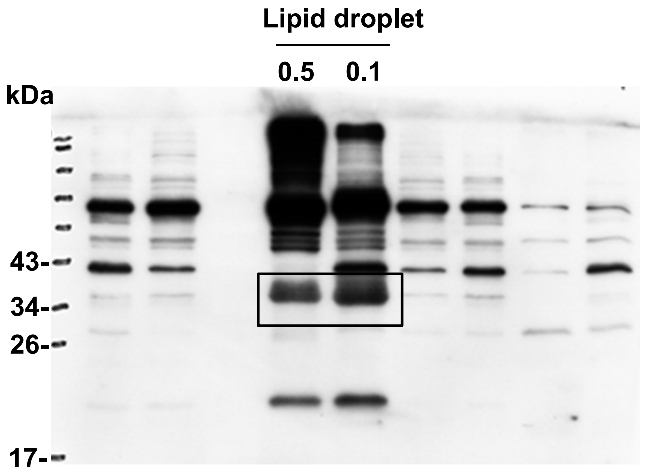


**Figure 4b**

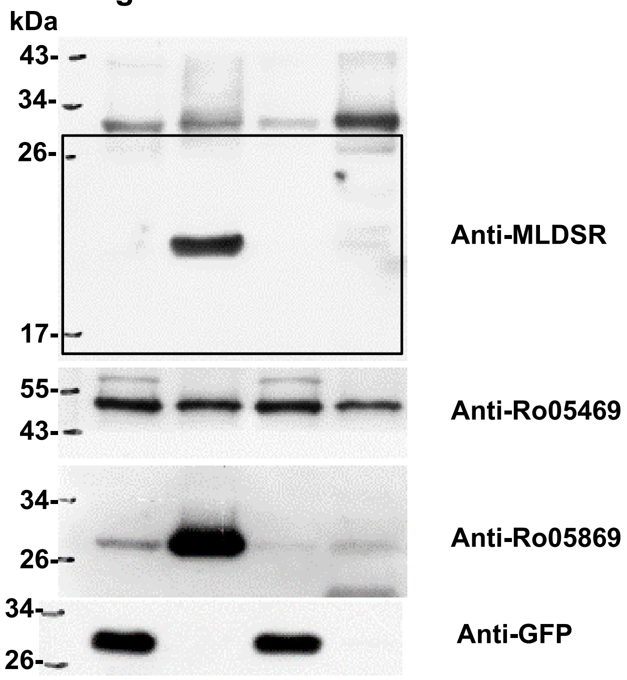


**Figure 4f** Anti-MLDS

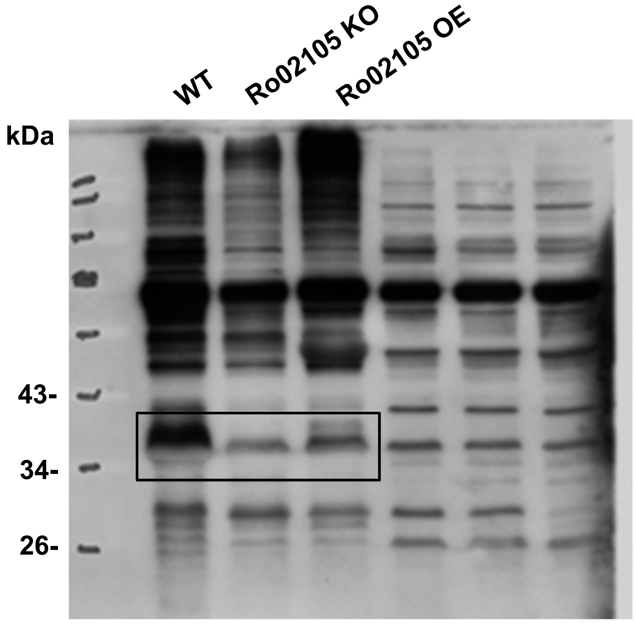


**Figure 7e** Anti-MLDSR

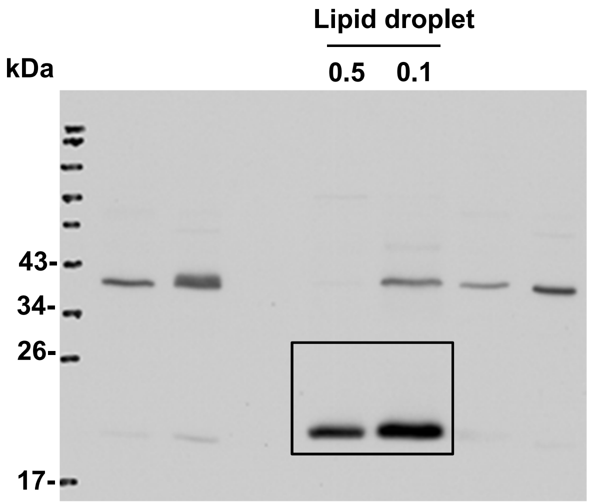

**Supplementary Figure 9: Original scans of the most important Western blots presented in the main figures.** The black boxes represent the cropped image in the main fugures.

**Supplementary Table 1: The putative DNA related proteins in RHA1 and PD630 LD proteome**

| Description | GI number/ Gene name |
|---|---|
| **RHA1** | |
| DNA topoisomerase I | 111021329 |
| DNA topoisomerase subunit A | 111020669 |
| DNA topoisomerase subunit B | 111020663 |
| DNA-directed RNA polymerase beta' subunit | 111018958 |
| DNA-directed RNA polymerase beta subunit | 111018959 |
| excision nuclease ABC subunit A | 111017983 |
| possible chromosome partitioning protein ParA | 111026081 |
| sigma factor, sigma 70 type, group 4 (ECF) | 111023159 |
| DNA-binding protein | 111023460 |
| DNA repair protein RecN | 111017951 |
| UvrABC system protein A | 111020821 |
| DNA-directed DNA polymerase I | 111018004 |
| DNA-directed DNA polymerase III alpha subunit | 111018082 |
| possible DNA polymerase III delta subunit | 111018296 |
| replicative DNA helicase | 111020417 |
| holliday junction DNA helicase | 111023845 |
| probable DNA repair helicase | 111021900 |
| probable helicase | 111018652 |
| probable chromosome partitioning protein | 111020643 |
| possible plasmid partitioning protein, ParB family | 111025381 |
| possible DNA binding protein | 111019018 |
| DNA-directed DNA replication initiator protein | 111020655 |
| probable DNA polymerase I | 111017852 |
| probable chromosome partitioning protein, ParA family | 111025382 |
| MLDS | 111019097 |
| JLP | 111017022 |
| **PD630** | |
| MLDS homolog | PD630_LPD06283 |
| UvrABC system protein A | PD630_LPD05054 |
| DNA-binding protein HU homolog | PD630_LPD03169 |
| Chromosomal replication initiator protein DnaA | PD630_LPD00001 |
| Probable chromosome-partitioning protein parB | PD630_LPD08045 |
| UvrABC system protein A | PD630_LPD00176 |
| Probable ATP-dependent DNA helicase recQ | PD630_LPD05408 |
| ATP-dependent DNA helicase pcrA | PD630_LPD02168 |
| DNA polymerase I | PD630_LPD05078 |
| DNA topoisomerase 1 | PD630_LPD00800 |
| DNA polymerase III subunit alpha | PD630_LPD05163 |
| ATP-dependent DNA helicase recG | PD630_LPD03186 |
| DNA gyrase subunit A | PD630_LPD00016 |
| DNA gyrase subunit B | PD630_LPD00009 |

**Supplementary Table 2: The proteins containing PAKKA motif in RHA1**

| Description | Max score | Total score | Query cover | E value | Ident | Gene name | Number of motif |
|---|---|---|---|---|---|---|---|
| **MLDS** | **17.6** | **70** | **100%** | **15** | **100%** | **RHA1_ro02104** | **4** |
| **JLP** | **17.6** | **324** | **100%** | **15** | **100%** | **RHA1_ro00689** | **7** |
| **DNA-binding protein** | **14.2** | **99** | **100%** | **162** | **100%** | **RHA1_ro06501** | **7** |
| **DNA topoisomerase I** | **17.6** | **109** | **100%** | **15** | **100%** | **RHA1_ro04353** | **3** |
| **cell division initiation protein** | **14.2** | **26** | **100%** | **162** | **100%** | **RHA1_ro01080** | **2** |
| nucleoid-structuring protein H-NS | 17.6 | 184 | 100% | 15 | 100% | RHA1_ro08479 | 8 |
| histone-like protein | 17.6 | 117 | 100% | 15 | 100% | RHA1_ro06984 | 5 |
| hypothetical protein | 15.1 | 74 | 100% | 89 | 100% | RHA1_ro07060 | 5 |
| histone H1 | 15.1 | 59 | 100% | 89 | 100% | RHA1_ro09106 | 4 |
| RNA polymerase sigma factor | 17.6 | 93 | 100% | 15 | 100% | RHA1_ro06823 | 2 |
| DNA binding protein, Ku-like | 17.6 | 47 | 100% | 15 | 100% | RHA1_ro07202 | 1 |
| hypothetical protein | 17.6 | 127 | 100% | 15 | 100% | RHA1_ro11088 | 3 |
| hypothetical protein | 17.6 | 18 | 100% | 15 | 100% | RHA1_ro00971 | 1 |
| hypothetical protein | 17.6 | 75 | 100% | 15 | 100% | RHA1_ro08235 | 1 |

Protein names in bold represent putative LD-associated proteins.
